# Enhancing Patient Support in Digital Inflammatory Bowel Disease Tools: The Need for Medication Guidance and Decision Support

**DOI:** 10.2196/82238

**Published:** 2025-09-26

**Authors:** Ali Sidat, Sian Uppal

**Affiliations:** 1 The Royal Wolverhampton NHS Trust, Wolverhampton Road, Heath Town, Wolverhampton, WV10 0QP, United Kingdom, 44 1902307999

**Keywords:** digital health, self-management, electronic medical records, quality of life, diet, mental health, inflammatory bowel disease, social determinants of health, implementation

With respect to the article “Developing and Evaluating a Bundled Digital Tool to Improve Complex Care and Self-Management of Patients With Inflammatory Bowel Disease: Protocol for a Hybrid Effectiveness-Implementation Study” [1], I wanted to raise a concern about one important area of patient support that I feel could be strengthened. While the protocol describes an app with potential to help patients manage their inflammatory bowel disease (IBD), I worry that its current design may not provide sufficient decision support in response to patient-reported symptoms.

As a training doctor with a special interest in IBD and digital health solutions, and as a former member of the young adults advisory board for a national IBD charity, I have seen the challenges from both a clinical and a patient advocacy perspective. In my medical training, I have encountered the complexity of modern IBD treatment, particularly the rapidly expanding use of biologics, and the barriers patients face in understanding and adhering to their medication plans. During my time on the advisory board, I listened to the experiences of young adults living with IBD who often felt overwhelmed by their treatment regimens, unsure where to find reliable information, and uncertain about what to do when symptoms changed.

These treatments are quite often complex. Biologics, immunomodulators, and combination therapies require strict adherence, careful monitoring, and awareness of possible side effects. While clinicians aim to cover these points in the clinic, patients may leave with only partial recall of what was discussed. Many turn to the internet for further information, where guidance can be inconsistent or inaccurate [2,3].

A thoughtfully designed IBD self-management app could close this gap by including a dedicated section tailored to each patient’s prescribed medications. This section could clearly explain the purpose of each drug, how it works, how and when to take it, and potential side effects. For biologics, in particular, clear instructions on injection schedules, the importance of monitoring blood tests, and what to do if a dose is missed would be especially valuable [2].

Beyond static information, the app could also incorporate responsive decision support. If a patient reports worsening abdominal pain, new bleeding, or unusual fatigue, the app could offer evidence-based prompts such as adjusting self-care, reviewing medication timing, or seeking prompt medical advice [2,4]. This would not replace clinical decision-making, but it could help prevent deterioration, avoid unnecessary emergency visits, and improve patient confidence in managing their condition [4,5].

If these features are not included, the tool risks falling short of its potential, leaving patients without the personalized guidance they need for increasingly complex regimens. On the other hand, integrating clear medication information and responsive symptom-based guidance could improve adherence, strengthen patient-clinician communication, and ultimately lead to better outcomes [4,5].

In summary, I urge the developers and researchers to consider embedding comprehensive, patient-specific medication guidance, particularly for biologics. Responsive decision support should also be considered in this promising tool. These additions would make it a more complete, patient-centered resource, better equipping people with IBD to manage their condition effectively.

## Abbreviations

IBD: inflammatory bowel disease
